# A new superfamily and family of the infraorder Hadziida (Amphipoda, Senticaudata) based on a new genus and species from the Clarion-Clipperton Zone, Pacific Ocean

**DOI:** 10.3897/zookeys.1274.140273

**Published:** 2026-03-24

**Authors:** Tammy Horton, Georgina Valls Domedel, Eva C. D. Stewart, Michael H. Thurston

**Affiliations:** 1 National Oceanography Centre, Southampton, SO14 3ZH, UK National Oceanography Centre Southampton United Kingdom; 2 School of Ocean and Earth Sciences, University of Southampton, Southampton, SO14 3ZH, UK School of Ocean and Earth Sciences, University of Southampton Southampton United Kingdom; 3 Life Sciences Department, Natural History Museum, London, Cromwell Road, South Kensington, SW7 5BD, UK Life Sciences Department, Natural History Museum London United Kingdom

**Keywords:** Abyss, amphipods, Clarion-Clipperton Zone, Hadziida, *Mirabestia* gen. nov., Mirabestiidae fam. nov., Mirabestioidea superfam. nov., Pacific Ocean, taxonomy

## Abstract

A remarkable new amphipod species, *Mirabestia maisie***sp. nov**., is described from the Clarion-Clipperton Zone, abyssal Pacific Ocean, at depths of 4130–4309 m. The species is described within the new taxa Mirabestioidea**superfam. nov**., Mirabestiidae**fam. nov**., and *Mirabestia***gen. nov**., within the suborder Senticaudata. Diagnoses and molecular sequences of five nuclear and mitochondrial genetic markers are provided for the new species. A preliminary phylogenetic reconstruction of the infraorder Hadziida is also presented, based on a concatenated dataset of the COI and histone 3 genetic markers.

## Introduction

The Hadziida Lowry & Myers, 2013 is one of six infraorders within the Senticaudata Lowry & Myers, 2013 and comprises 209 genera and 1170 species (data extracted from Horton et al. 2024). The infraorder includes three superfamilies, Calliopioidea Sars, 1893, Hadzioidea S. Karaman, 1943 and Magnovioidea Alves, Lowry & Johnsson, 2020. The first two groups are speciose, geographically widespread, and largely shallow-water marine in occurrence, although both include freshwater species. The Calliopioidea comprises five families, 69 genera, and 328 species, and the Hadzioidea comprises 8 families, 139 genera, and 841 species (data extracted from Horton et al. 2024). The Magnovioidea is represented by a single genus and species collected at shelf depths on the Brazilian coast (Alves et al. 2020).

The new species described here is very distinct in terms of its morphology but is also found much deeper than most other hadziids. Approximately 20% of Hadziida species are confined to brackish or freshwater habitats. A further 70% of species occur at shelf depths of less than 200 m, but the species described herein is only the eighth (<1%) to have been recorded at depths exceeding 4000 m (unpublished data). The seven hadziid species reported deeper than 4000 m are: *Harpinioides fissicauda* (Schellenberg, 1926) (4893 m; Barnard 1962); *Bathyschraderia fragilis* Kamenskaya, 1981 (7000–9990 m); *Bathyschraderia magnifica* Dahl, 1959 (6960–7000 m); *Bathyceradocus stephenseni* Pirlot, 1934 (7250–7290 m; Dahl 1959); *Bathyceradocus hawkingi* Jażdżewska & Ziemkiewicz, 2019 (5217–5229 m), *Bathyceradocus iberiensis* Andres, 1977 (5315 m); and *Metaceracoides vitjazi* Birstein & N. Vinogradova, 1960 (7190–8900 m; Kamenskaya 1981).

The spination of the uropods indicate placement within the suborder Senticaudata, but the possession of conical mouthparts is unique within this group. Characters of the taxa reported here do not conform with those of any of the hadziid superfamilies. This study therefore adds a remarkable new superfamily, Mirabestioidea, new family Mirabestiidae, and new genus and species, collected from the Clarion-Clipperton Zone in the Pacific Ocean at depths of 4130–4309 m. We provide diagnoses of the new taxa, and a description and illustrations of the new species. We provide molecular sequence data for two mitochondrial and three nuclear genetic markers for a large number of specimens of the new species both as an aid in identification and as a starting point for future evolutionary analysis. A preliminary phylogenetic reconstruction of the infraorder Hadziida is also presented, based on a concatenated dataset of the cytochrome c-oxidase subunit I and histone 3 genetic markers, from comparative taxa with these sequences available in GenBank.

## Methods

The material for the present study was sampled in the central-east Pacific Ocean, specifically in the easternmost sector of the Clarion-Clipperton Zone (CCZ). The material studied was collected using an USNEL spade box corer (Ocean Instruments BX-650; BC) and epibenthic sledge (EBS) during seven expeditions to two different exploration contract areas (henceforth, contract areas) in the CCZ, five cruises to the NORI-D (Nauru Ocean Resources Inc.) contract area (C5A in 2020; C5D in 2021; C7A & C7B in 2022; and 8A in 2023) following methods in Glover et al. (2015), and two to the BGR (Bundesanstalt für Geowissenschaften und Rohstoffe) contract area (MANGAN 2018 (Rühlemann et al. 2019) and MANGAN 2023 (Rühlemann et al. 2023)) (Table 1). For details of gear types, deployment and sample processing see Jażdżewska and Horton (2026).

**Table 1.** Station data.

**Claim ID**	**Vessel**	**Cruise**	**Station**	**Gear**	**Latitude (N), Longitude (W)**	**Depth (m)**	**Date**
NORI-D	RV Maersk Launcher	5A	SWM_020	BC_366	10.3984°N, 117.2723°W	4328	16/11/2020
NORI-D	RV Maersk Launcher	5A	STM_012	BC_370	10.3789°N, 117.1535°W	4305	17/11/2020
NORI-D	RV Maersk Launcher	5A	STM_047	BC_377	10.3361°N, 117.2050°W	4284	22/11/2020
NORI-D	RV Maersk Launcher	5A	STM_050	BC_379	10.3549°N, 117.2208°W	4273	23/11/2020
NORI-D	RV Maersk Launcher	5D	STM_170	BC_386	10.3675°N, 117.1544°W	4305	11/05/2021
NORI-D	RV Maersk Launcher	5D	STM_176	BC_391	10.3218°N, 117.1975°W	4280	13/05/2021
NORI-D	RV Maersk Launcher	5D	STM_168	BC_393	10.3784°N, 117.1441°W	4309	14/05/2021
NORI-D	RV Maersk Launcher	5D	STM_167	BC_394	10.3777°N, 117.1340°W	4305	14/05/2021
NORI-D	RV Maersk Launcher	5D	STM_175	BC_399	10.3546°N, 117.1532°W	4290	16/05/2021
NORI-D	RV Maersk Launcher	5D	STM_174	BC_402	10.3481°N, 117.1707°W	4282	17/05/2021
NORI-D	RV Maersk Launcher	5D	SPR_160	BC_412	10.8397°N, 116.1504°W	4132	25/05/2021
NORI-D	MV Island Pride	7A	TF_005	BC_427	10.3325°N, 117.1874°W	4281	24/08/2022
NORI-D	MV Island Pride	7A	OTF_008	BC_449	10.3539°N, 117.1713°W	4273	09/09/2022
NORI-D	MV Island Pride	7B	STM_189	BC_460	10.3509°N, 117.2485°W	4293	19/11/2022
NORI-D	MV Island Pride	7B	TF025_03	BC_465	10.3341°N, 117.1772°W	4282	22/11/2022
NORI-D	MV Island Pride	7B	TF025_04	BC_466	10.3342°N, 117.1771°W	4282	22/11/2022
NORI-D	MV Island Pride	7B	OTF_008	BC_468	10.3537°N, 117.1714°W	4250	26/11/2022
NORI-D	MV Island Pride	7B	STM_173	BC_471	10.3573°N, 117.1698°W	4273	27/11/2022
NORI-D	MV Coco	8A	IM002_BC	BC_490	10.3597°N, 117.2445°W	4290	25/11/2023
NORI-D	MV Coco	8A	IM004_BC	BC_502	10.3549°N, 117.2459°W	4289	14/12/2023
BGR area	RV Sonne	MANGAN 2018	SO 262-155	EBS	11.7910°N, 117.5370°W	4352	09/05/2018
BGR area	RV Kilo Moana	MANGAN 2023	KM23-7	EBS	12.0380°N, 138.0920°W	5109	17/04/2023

Specimens were dissected and mounted on permanent slides using polyvinyl-lactophenol stained with lignin pink. Illustrations were made using Leica M125 and Olympus BX53 microscopes. Pencil drawings were scanned and inked digitally using Adobe Illustrator and a WACOM digitiser tablet (Coleman 2003, 2009). Some setae were omitted from the illustrations for clarity. Appendages of the right side were dissected and illustrated, unless otherwise stated.

The following abbreviations were used: **A1, 2** = antenna 1, 2; **C1–5** = coxa 1–5; **Ep** = epistome; **G1, 2** = gnathopod 1, 2; **H** = head; **LL** = lower lip; **Md** = mandible; **Mx1, 2** = maxilla 1, 2; **Mxp** = maxilliped; **P3–7** = pereopod 3–7; **T** = telson; **U1–3** = uropod 1–3; **UL** = upper lip; **l** = left; **r** = right.

Type material is deposited in the Natural History Museum, London (**NHMUK**). Additional material is kept in the Deutsches Zentrum für Marine Biodiversitätsforschung (DZMB) in Wilhelmshaven and the Discovery Collections at the National Oceanography Centre, Southampton (**DISCOLL**).

### DNA extraction, amplification, and sequencing

Specimens collected from MANGAN cruises were extracted and sequenced as described in Jażdżewska et al. (2025). Specimens collected from the NORI–D area were processed as follows.

DNA was extracted from a pair of pleopods using QuickExtract^TM^ DNA extraction solution (Lucigen), following manufacturer guidelines, and adapted for a digestion time of 45 minutes. Regions of two mitochondrial [16S rRNA (16S) and cytochrome c-oxidase subunit I (COI)] and three nuclear [28S rRNA (28S), 18S rRNA (18S), and early-stage histone 3 (H3)] genetic markers were amplified with published primer sets (Medlin et al. 1988; Nygren and Sundberg 2003; Astrin and Stüben 2008; Corrigan et al. 2014; Lörz et al. 2018). The PCR mix for each reaction contained 10.5 µl of Red Taq DNA Polymerase 1.1X MasterMix (VWR), 0.5 µl of each primer (10 µM), and 1 µl of DNA template. Primers and PCR conditions are detailed in Table 2, COI PCR protocol was followed from Witt et al. (2006). The primers used for sequencing were the same as those for amplifications, with an additional set of internal primers for 18S: 620F (TAAAGYTGYTGCAGTTAAA; Nygren and Sundberg 2003) and 1324R (CGGCCATGCACCACC; Cohen et al. 1998). PCR products were purified using a Millipore Multiscreen 96-well PCR Purification System and sequenced using an ABI 3730XL DNA Analyzer (Applied Biosystems) at The Natural History Museum Sequencing Facilities. For each gene fragment contigs were assembled by aligning both forward and reverse sequences, chromatograms were visually inspected, and ambiguous base calls were corrected manually, using Geneious v. 7.0.6 (Kearse et al. 2012).

**Table 2.** Primers and PCR programs used for DNA amplification.

**Gene**	**Primer**		**Sequence (5' – 3')**	**PCR program**	**Reference**
COI	LCO1490-JJ	Forward	CHACWAAYCATAAAGATATYGG	1× (2 min at 94 °C), 5× (30 s at 94 °C, 90 s at 45 °C, 60 s at 72 °C), 35× (30 s at 94 °C, 90 s at 51 °C, 60 s at 72 °C), 1× (5 min at 74 °C)	Astrin and Stüben 2008
	HCO2198-JJ	Reverse	AWACTTCVGGRTGVCCAAARAATCA		Astrin and Stüben 2008
16S	16SFt_amp	Forward	GCRGTATIYTRACYGTGCTAAGG	1× (2 min at 95 °C), 35× (30 s at 95 °C, 30 s at 50 °C, 45 s at 72 °C), 1× (5 min at 72 °C)	Lörz et al. 2018
	16SRt_amp	Reverse	CTGGCTTAAACCGRTYTGAACTC		Lörz et al. 2018
28S	28Sftw	Forward	AGGCGGAATGTTGCGT	1× (2 min at 95 °C), 35× (40 s at 94 °C, 40 s at 50 °C, 40 s at 72 °C), 1× (10 min at 72 °C)	Corrigan et al. 2014
	28Srtw	Reverse	CTGAGCGGTTTCACGGTC		Corrigan et al. 2014
18S	18SA	Forward	AYCTGGTTGATCCTGCCAGT	1× (5 min at 95 °C), 30× (30s at 95 °C, 30 s at 59 °C, 60 s at 72 °C), 1× (10 min at 72 °C)	Medlin et al. 1988
	18SB	Reverse	ACCTTGTTACGACTTTTACTTCCTC		Nygren and Sundberg 2003
H3	HisH3f	Forward	AAATAGCYCGTACYAAGCAGAC	1× (2 min at 95 °C), 35× (40 s at 94 °C, 40 s at 45 °C, 40s at 72 °C), 1× (10 min at 72 °C)	Corrigan et al. 2014
	HisH3r	Reverse	ATTGAATRTCYTTGGGCATGAT		Corrigan et al. 2014

All sequences are deposited in GenBank with the accession numbers as reported in Table 4. For specimens collected during MANGAN cruises, voucher information, taxonomic classifications and sequences are deposited in the data set “DS-AMPHICCZ” in the Barcode of Life Data System (BOLD) (https://doi.org/10.5883/DS-AMPHICCZ) (www.boldsystems.org) (Ratnasingham and Hebert 2007).

### Phylogenetic analysis

Phylogenetic reconstruction of the infraorder Hadziida was conducted using a concatenated dataset of COI and H3 genetic sequences, as these markers had the highest taxonomic coverage available across the infraorder. Newly obtained sequences were added to a dataset of COI and H3 sequences from species across the superfamilies Hadzioidea and Calliopioidea available on GenBank (Table 3). Two outgroup taxa were selected from the superfamily Gammaroidea. Sequences were aligned using MUSCLE (Edgar 2004) in Geneious v. 7.0.6; with nucleotides translated into amino acids to identify pseudogenes based on the presence of stop codons. Ambiguously aligned regions were filtered using the Gblocks server (http://phylogeny.lirmm.fr/phylo_cgi/one_task.cgi?task_type=gblocks), allowing gap positions in final blocks and less strict flanking positions. The final sequence alignment used for analyses was 982 bp. The optimal substitution model for each gene was determined using IQTREE ModelFinder (Kalyaanamoorthy et al. 2017). This was identified as TIM + F + I + G4 for COI and TIM2 + G4 for H3.

**Table 3.** GenBank accession numbers for comparative sequences of COI and H3 downloaded and used in phylogenetic analyses. Outgroup taxa are noted with an asterisk (*). Taxonomic authorities can be found on the World Amphipoda Database (Horton et al. 2024).

**Species**	**COI**	**H3**	**Reference**
*Abludomelita japonica*	LC637611	LC637975	Tomikawa et al. 2022
*Abludomelita klitinii*	LC637599	LC637963	Tomikawa et al. 2022
*Awacaris japonica*	LC146846	LC334124	Tomikawa et al. 2017; Nakano and Tomikawa 2018
*Awacaris rhyaca*	LC146858	LC334127	Tomikawa et al. 2017; Nakano and Tomikawa 2018
*Awacaris yezoensis*	LC146845	LC334120	Tomikawa et al. 2017; Nakano and Tomikawa 2018
*Calliopius ezoensis*	LC512906	LC334137	Nakano and Tomikawa 2018; Shimoji et al. 2020
*Dulichiella tomioka*	LC637612	LC637976	Tomikawa et al. 2022
*Elasmopus nkjaf*	LC215813	LC215815	Nakamura et al. 2019
*Gammarella cyclodactyla*	LC637613	LC637977	Tomikawa et al. 2022
*Melita choshigawaensis*	LC371923	LC637946	Tomikawa et al. 2022
*Melita nagatai*	LC637584	LC637947	Tomikawa et al. 2022
*Melita okinawaensis*	LC637583	LC637945	Tomikawa et al. 2022
*Melita setiflagella*	LC637585	LC637948	Tomikawa et al. 2022
*Melita shimizui*	LC371926	LC637943	Tomikawa et al. 2022
*Metacrangonyx ilvanus*	HE967181	FR846131	Bauza-Ribot et al. 2011; Bauza-Ribot et al. 2012
*Paramoera erimoensis*	LC146874	LC334141	Tomikawa et al. 2017; Nakano and Tomikawa 2018
*Paramoera koysama*	MK358135	LC334140	Nakano and Tomikawa 2018; Sidorov 2020
*Paramoera shakotanensis*	LC146871	LC334142	Tomikawa et al. 2017; Nakano and Tomikawa 2018
*Pontogeneia rostrata*	JX545475	LC334145	Best and Stachowicz 2013; Nakano and Tomikawa 2018
*Tegano shiodamari*	LC637586	LC637950	Tomikawa et al. 2022
*Victoriopisa ryukyuensis*	LC637615	LC637979	Tomikawa et al. 2022
Amathillina cristata*	ON258084	OP697041	Copilas-Ciocianu et al. 2022; Copilas-Ciocianu et al. 2023
Gammarus aequicauda*	AY926667	OM827512	MacDonald et al. 2005; Hou et al. 2022

**Table 4.** Material examined and genetic sequence information. n.d. = not determined owing either to damaged specimen or specimen too small to determine.

**Specimen Number**	**Type Status**	**Sex**	**Size (mm)**	**Catalogue Number**	**Sample / Station Number**	**16S**	**COI**	**28S**	**H3**	**18S**
***Mirabestia maisie* sp. nov**.										
6179_TH_AMP_1	Paratype	immature	5.06	NHMUK 2025.34	BC_366		PV077116			
6592_TH_AMP_1	Other	n.d.		DISCOLL-TMC-AMP-6592-a	BC_377					
6759_TH_AMP_1	Other	n.d.		DISCOLL-TMC-AMP-6759-a	BC_386		PV077118			
6976_TH_AMP_1	Holotype	immature	8.03	NHMUK 2025.33	BC_391		PV077119		PV078014	
7092_TH_AMP_1	Other	immature		DISCOLL-TMC-AMP-7092-a	BC_393			PV075060		
7172_TH_AMP_1	Other	n.d.		DISCOLL-TMC-AMP-7172-a	BC_394					
7445_TH_AMP_1	Paratype	female	7.45	NHMUK 2025.35	BC_399			PV075061	PV078015	
7507_TH_AMP_1	Other	n.d.		DISCOLL-TMC-AMP-7507-a	BC_399					
7507_TH_AMP_2	Other	n.d.		DISCOLL-TMC-AMP-7507-b	BC_399					
7673_TH_AMP_1	Other	female	5.42	DISCOLL-TMC-AMP-7673-a	BC_402		PV077120	PV075062		
8222_TH_AMP_1	Other	male	6.71	DISCOLL-TMC-AMP-8222-a	BC_412		PV077121	PV075063		
8788_TH_AMP_1	Other	immature	5.48	DISCOLL-TMC-AMP-8788-a	BC_370			PV075064	PV078016	
8812_TH_AMP_1	Other	n.d.	4.51	DISCOLL-TMC-AMP-8812-a	BC_379			PV075065	PV078017	
9346_TH_AMP_2	Other	n.d.		DISCOLL-TMC-AMP-9346-b	BC_449			PV075066	PV078018	
9396_TH_AMP_1	Paratype	male	6.46	NHMUK 2025.36	BC_427	PV075081	PV077122	PV075067	PV078019	
9775_TH_AMP_1	Paratype	male	6.41	NHMUK 2025.37	BC_465	PV075082	PV077123	PV075068	PV078020	
10347_TH_AMP_1	Paratype	immature	4.2	NHMUK 2025.38	BC_471					
10363_TH_AMP_1a	Other	n.d.		DISCOLL-TMC-AMP-10363-a	BC_460					
10363_TH_AMP_1b	Other	n.d.		DISCOLL-TMC-AMP-10363-b	BC_460					
10385_TH_AMP_1	Other	n.d.		DISCOLL-TMC-AMP-10385-a	BC_466	PV075083	PV077124	PV075069	PV078021	PV077020
10390_TH_AMP_3	Other	n.d.		DISCOLL-TMC-AMP-10390-c	BC_468					
12542_TH_AMP_2	Other	n.d.		DISCOLL-TMC-AMP-12542-b	BC_490					
12551_TH_AMP_1	Other	n.d.		DISCOLL-TMC-AMP-12551-a	BC_502		PV077125			
DSB_3631	Other	n.d.		DSB_3631	SO 262-155		PQ734360			
DSB_3637	Other	n.d.		DSB_3637	SO 262-155		PQ734538			
DSB_7857	Other	n.d.		DSB_7857	KM23-7		PQ734548			
***Mirabestia* sp. [NHM_6660]**										
6660_TH_AMP_1	Other	immature	6.76	DISCOLL-TMC-AMP-6660-a	BC_379	PV075080	PV077117	PV075059	PV078013	

Phylogenetic trees were estimated using both Bayesian inference (BEAST v. 2.4.7; Bouckaert et al. 2019) and a maximum-likelihood approach implemented with IQ-TREE v. 2.2.6. (Minh et al. 2020). Node support in IQ-TREE was estimated using 1000 ultrafast bootstraps (Hoang et al. 2018). BEAST analyses were performed with trees and clock models linked, a Yule tree model, and a relaxed clock log normal. Two independent runs of a maximum of 30 million steps were combined after discarding 10% as burn-in. Runs were checked for convergence (ESS > 200) and a median consensus tree was estimated from the combined post-burn-in samples.

## Results

### Systematics

**Order Amphipoda Latreille, 1816**

**Suborder Senticaudata Lowry & Myers, 2013**

**Infraorder Hadziida Lowry & Myers, 2013**

**Parvorder Hadziidira Lowry & Myers, 2013**

#### Mirabestioidea

Taxon classificationAnimaliaAmphipodaMirabestiidae

super fam. nov.

16966634-2A28-5013-B213-3ECD49F3E5D4

https://zoobank.org/20A054F0-90C2-467E-81A5-CC42A0877C80

##### Diagnosis.

Body compressed. ***Antenna 1 longer than antenna 2***. Antenna 2 article 1 not bulbous. Eyes absent. ***Mouthparts conical***. Mandible molar medium. Maxilliped outer plate as long as palp article 3. Coxal gills on pereonite 2–6. ***Gnathopods disparate*. *Gnathopod 1 simple***. Gnathopod 2 subchelate, ***less broad than gnathopod 1***. Pleonites 2–3, urosomites 1–2 multidentate on posterodorsal margin. Urosomite 2 lacking dorsal concavity and enclosed setae. Uropod 1 lacking basofacial setae. Uropod 3 biramous, peduncle and rami of equal length, rami subequal in length, fringed with long plumose setae, outer ramus with minute second article. Telson minute, deeply cleft. (***Highlighted*** characters separate the Mirabestoidea from the other superfamilies).

##### Included families.

Mirabestiidae fam. nov.

##### Remarks.

The combination of conical mouthparts, disparate gnathopods and simple gnathopod 1 is unique within the infraorder Hadziida. Despite not fully conforming to the diagnosis of the Hadziida in Lowry and Myers (2013), this taxon presents a general morphology that is closer to this infraorder than to the other senticaudate infraorders. As indicated by Lowry and Myers (2017: 10), “In determining synapomorphies we record a character state as synapomorphic if it is present in the majority, not necessarily all, of the members of a family or higher taxon”, and some of the families within the infraorder do not have all of the diagnostic characters in all genera.

**Figure 1.**
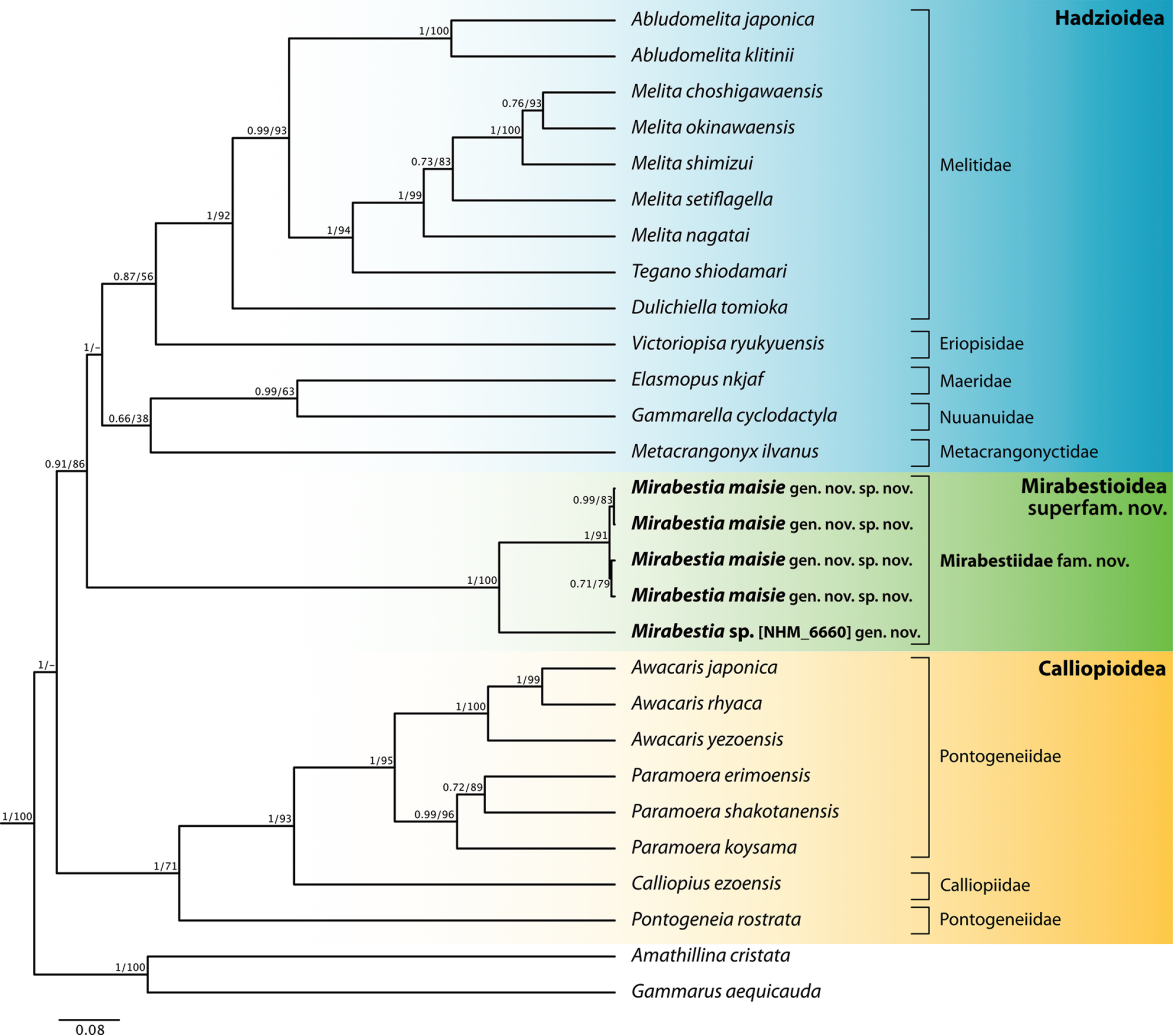
Bayesian ultrametric tree showing the hypothesised relationships between species across the Hadziida, based on a concatenated dataset of available COI and H3 sequence data. Bayesian posterior probability values (before /) and maximum-likelihood bootstrap values (after /) are shown on branch nodes. Family and superfamily are denoted on the right.

*Mirabestia* gen. nov. conforms to the diagnostic description of the Hadziida in Alves et al. (2020). It is noted that in their diagnosis the uropod 1 basofacial setae are indicated as present or absent (absent in *Mirabestia* gen.nov.). Molecular phylogenetic analysis also supports the erection of a new superfamily (Fig. 1). Based on COI and H3 sequence data, Mirabestioidea superfam. nov. forms a well-supported monophyletic clade (BI = 0.91, ML = 86) which is hypothesised as sister to Hadzioidea, with Calliopioidea as the basal superfamily within the infraorder Hadziida, although it is recognised that the number of taxa used in this analysis is low and that the confidence in such analyses will be improved only by the inclusion of a greater number of comparative taxa and more genetic markers. Although 16S, 18S, and 28S were sequenced for a number of specimens of *Mirabestia maisie* sp. nov. there were few/no taxa available in GenBank which also had these sequences available.

#### Mirabestiidae fam. nov.

Taxon classificationAnimaliaAmphipodaMirabestiidae

18C1F4FC-CA40-5F07-B8D5-05C8A316F75B

https://zoobank.org/875CD4BA-7FA0-4CDC-9D17-3793BB7FE8AF

##### Diagnosis.

With the characters of the superfamily.

##### Etymology.

The family name is based on the type genus *Mirabestia*.

##### Remarks.

Conical mouthparts with a piercing and suctorial function are known to occur in genera within the Acanthonotozomatidae Stebbing, 1906, Didymocheliidae Bellan-Santini & Ledoyer, 1987, Iphimediidae Boeck, 1871, Ochlesidae Stebbing, 1910, Pardaliscidae Boeck, 1871, and Sicafodiidae Just, 2004 and as a minority trend among various genera within the Lysianassoidea Dana, 1849 (e.g. *Acidostoma* Lilljeborg, 1865), but hitherto they have not been recorded in any senticaudate taxon.

#### Mirabestia gen. nov.

Taxon classificationAnimaliaAmphipodaMirabestiidae

FD9A49AC-4F95-5811-97AE-32175A27F8EA

https://zoobank.org/3426B2D6-9FAF-426C-A50F-CB2D50F29623

##### Type species.

*Mirabestia maisie* sp. nov. (by monotypy).

##### Etymology.

The name *Mirabestia* (feminine) is derived from Latin *mirus* meaning “wonderful” or “extraordinary”; combined with *bestia* meaning “beast”, in reference to the wonderful and extraordinary morphology exhibited by this beast.

##### Generic diagnosis.

With the characters of the superfamily.

#### Mirabestia maisie sp. nov.

Taxon classificationAnimaliaAmphipodaMirabestiidae

50BD26B6-F9B8-5B83-89AA-3253F505F303

https://zoobank.org/32D73CC4-04B6-4D72-B99B-189ABADEA891

**Figure 2.**
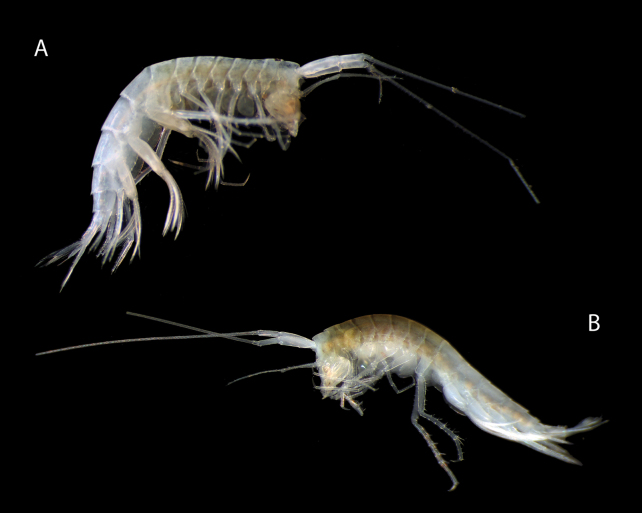
**A***Mirabestia maisie* sp. nov. immature, 8.0 mm, holotype: NHMUK 2025.33, habitus, fresh specimen **B***Mirabestia maisie* sp. nov. mature female, 7.45 mm, paratype, NHMUK 2025.35, habitus, fresh specimen.

**Figure 3.**
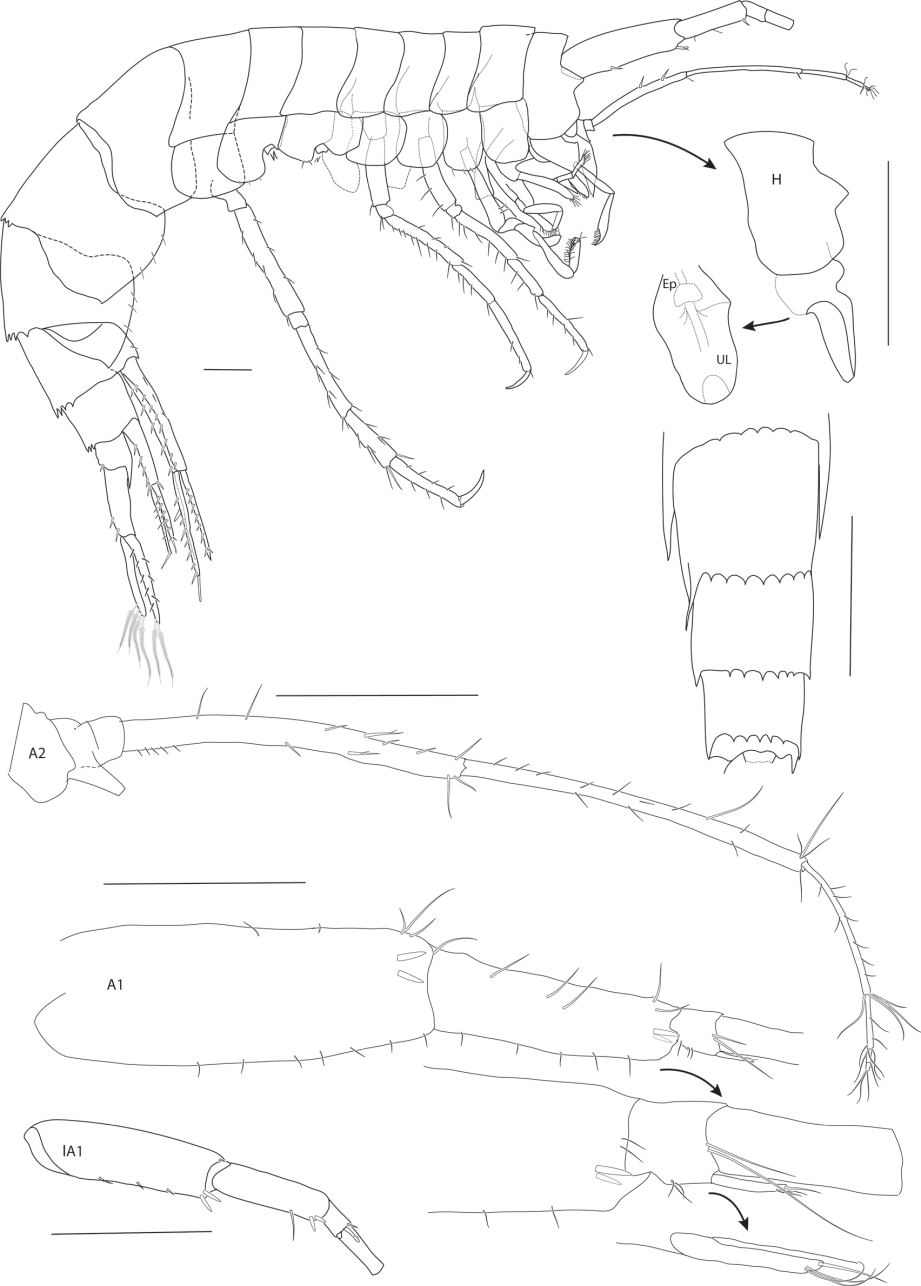
*Mirabestia maisie* sp. nov. immature, 8.0 mm, holotype, NHMUK 2025.33. Scale bars: 0.5 mm (Habitus, A1, 2), 1 mm (H, UL, Ep, lA1).

**Figure 4.**
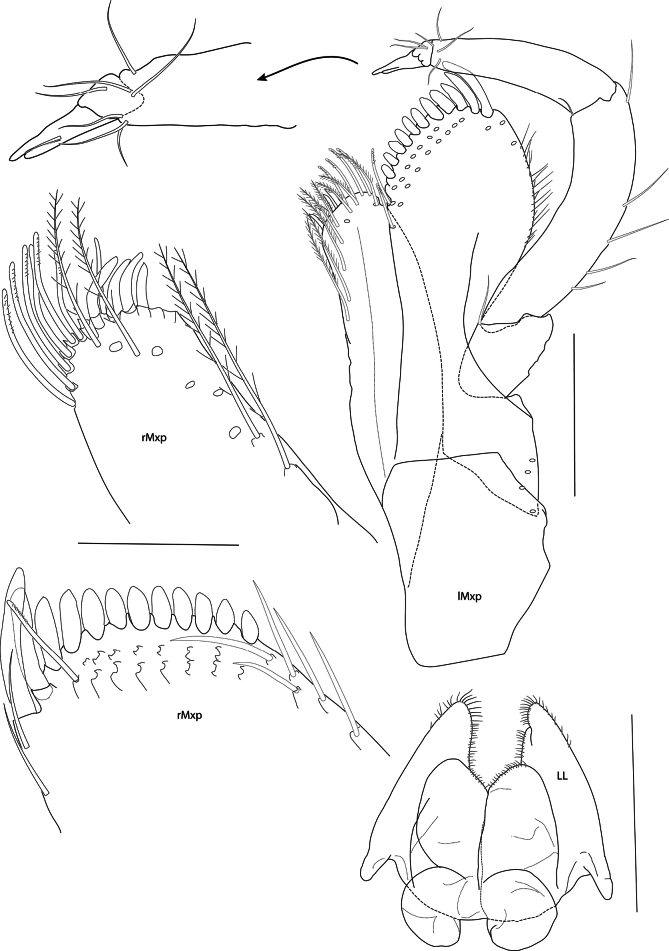
*Mirabestia maisie* sp. nov. immature, 8.0 mm, holotype, NHMUK 2025.33. Scale bars: 0.2 mm (lMxp), 0.5 mm (LL), 0.1 mm (rMxp detail).

**Figure 5.**
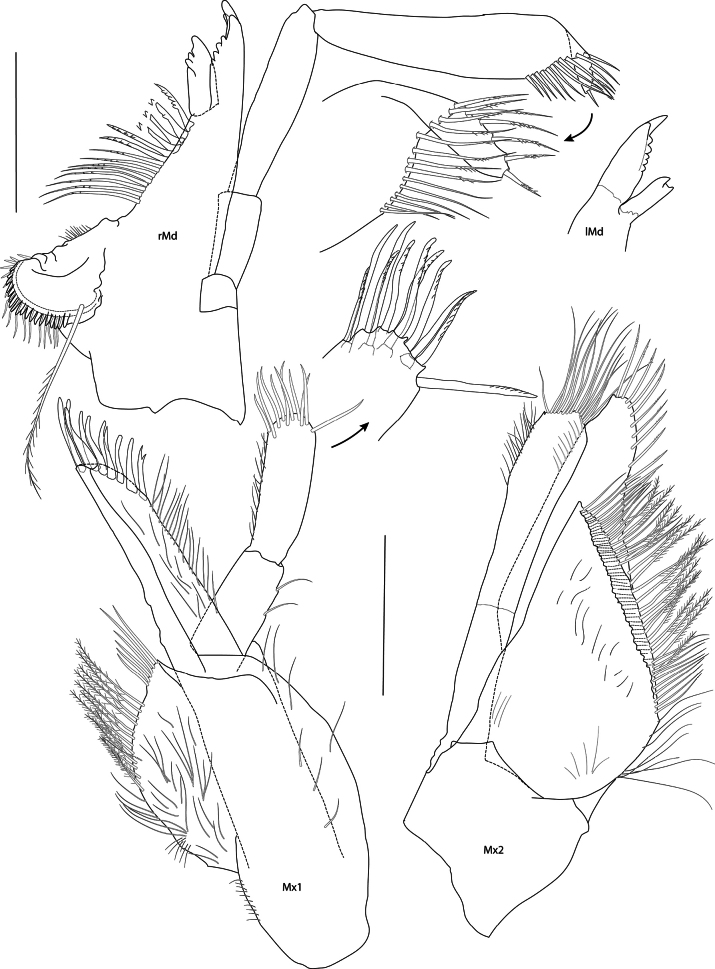
*Mirabestia maisie* sp. nov. immature, 8.0 mm, holotype, NHMUK 2025.33. Scale bars: 0.2 mm (Mx1–2, Md).

**Figure 6.**
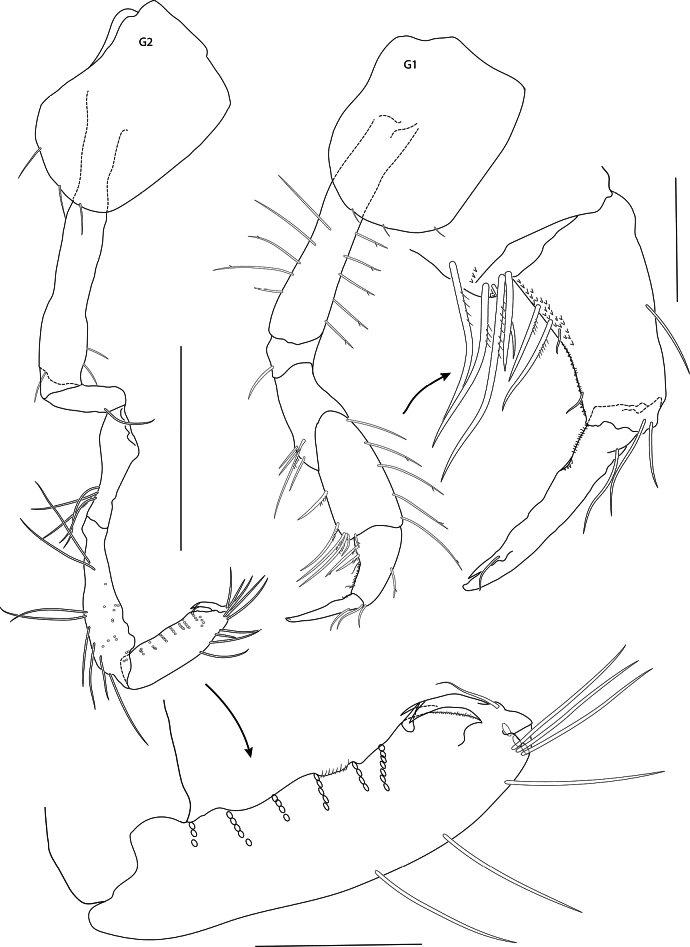
*Mirabestia maisie* sp. nov. immature, 8.0 mm, holotype, NHMUK 2025.33. Scale bars: 0.5 mm (G1–2), 0.1 mm (detail).

**Figure 7.**
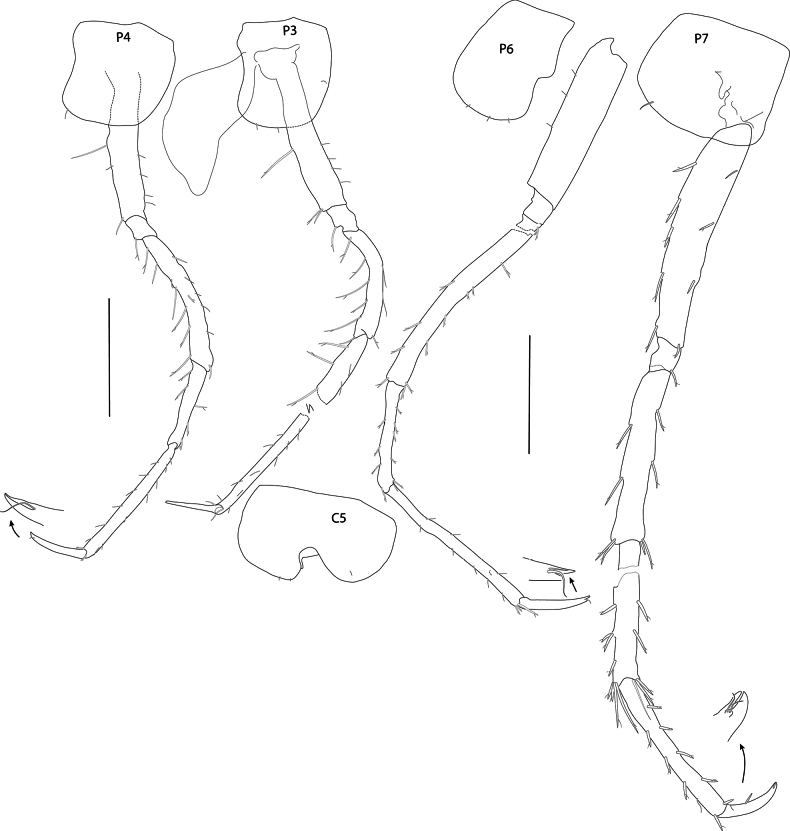
*Mirabestia maisie* sp. nov. immature, 8.0 mm, holotype, NHMUK 2025.33. Scale bars: 0.5 mm (P3–7).

**Figure 8.**
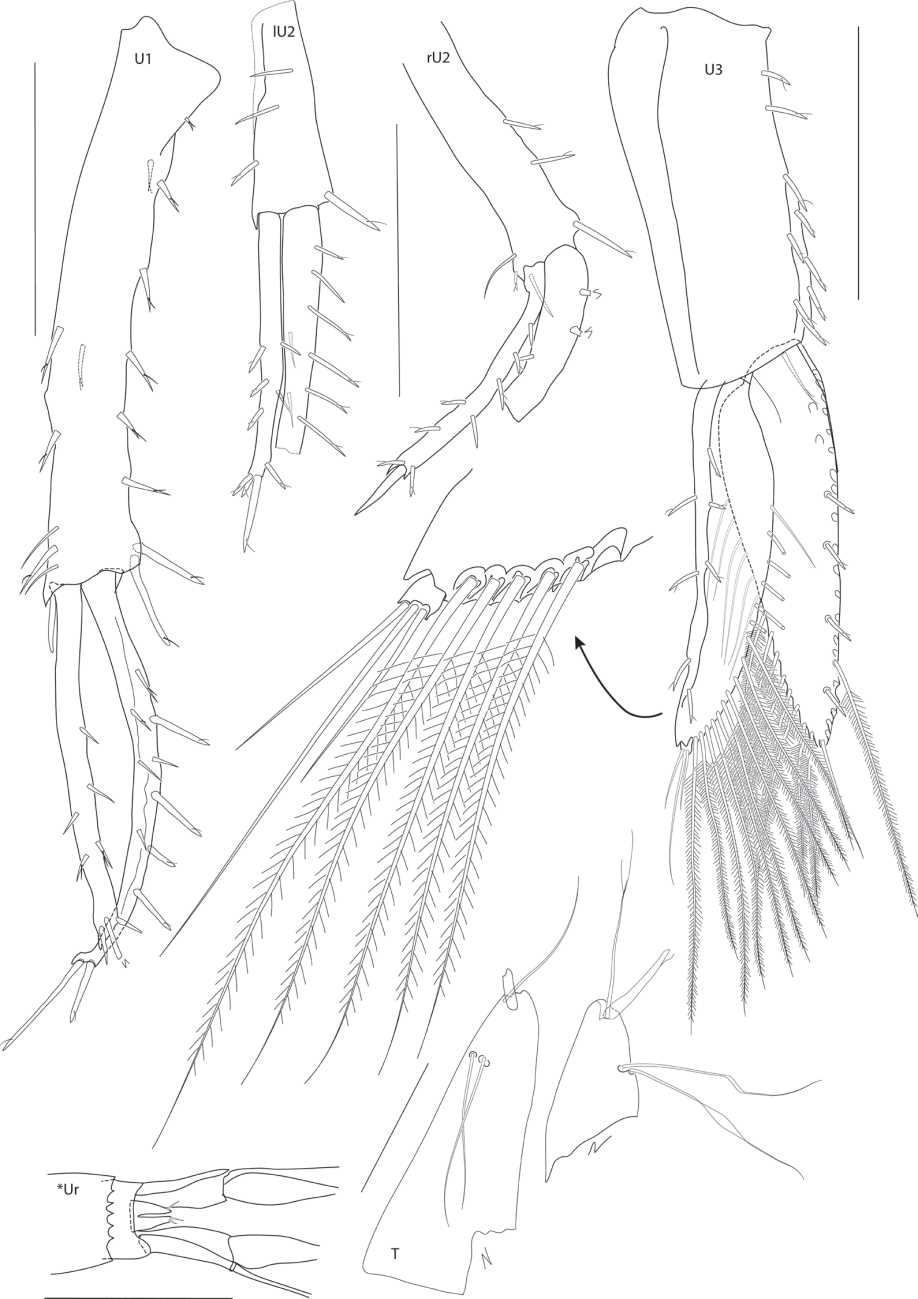
*Mirabestia maisie* sp. nov. immature, 8.0 mm, holotype, NHMUK 2025.33. Scale bars: 0.5 mm (U1–U3); 0.1 mm (T). *Ur = Urosomite 2, 3 & Telson of paratype NHMUK 2025.35. Scale bar: 1 mm.

Figs 2, 3, 4, 5, 6, 7, 8

##### Type material.

***Holotype***: Pacific • immature (no penes or oostegites apparent), 8.0 mm; carcass and 19 slides; Clarion-Clipperton Zone; 10.32°N, 117.20°W; depth 4280 m; 13/05/2021; NORI-D contract area, RV Maersk Launcher, Cruise 5D, Station STM_176, Box Core BC_391; NHMUK 2025.33 (specimen 6976_TH_AMP1); COI (PV077119), H3 (PV077114).

***Paratypes***: Pacific • mature female with brood (5 eggs), 7.45 mm; Clarion-Clipperton Zone; 10.35°N, 117.15°W; depth 4290 m; 16/05/2021; NORI-D contract area, RV Maersk Launcher, Cruise 5D, Station STM_175, Box Core BC_399; NHMUK 2025.35 (specimen 7445_TH_AMP_1); 28S (PV075061), H3 (PV078015) • male (penes apparent), 6.46 mm; Clarion-Clipperton Zone; 10.33°N,117.19°W; depth 4281 m; 24/08/2022; NORI-D contract area, MV Island Pride, Cruise 7A, Station TF_005, Box Core BC_427; NHMUK 2025.36 (specimen 9396_TH_AMP_1); COI (PV077122), 16S (PV075081), 28S (PV075067), H3 (PV078019) • male (penes apparent), 6.41 mm; Clarion-Clipperton Zone; 10.33°N,117.18°W; depth 4282 m; 22/11/2022; NORI-D contract area, MV Island Pride, Cruise 7B, Station TF025_03, Box Core BC_465; NHMUK 2025.37 (specimen 9775_TH_AMP_1); COI (PV077123), 16S (PV075082), 28S (PV075068), H3 (PV078020) • immature (no oostegites or penes visible), 4.2 mm; Clarion-Clipperton Zone; 10.36°N, 117.17°W; 4273 m; 27/11/2022; NORI-D contract area, MV Island Pride, Cruise 7B, Station STM_173, Box Core BC_471; NHMUK 2025.38 (specimen 10347_TH_AMP_1) • immature (no oostegites or penes visible), 5.06 mm; Clarion-Clipperton Zone; 10.40°N, 117.27°W; depth 4328 m; 16/11/2020; NORI-D contract area, RV Maersk Launcher, Cruise 5A, Box Core BC_366; NHMUK 2025.34 (specimen 6179_TH_AMP); COI (PV077116).

##### Other material.

Pacific • Clarion-Clipperton Zone; NORI-D and BGR contract areas, several stations (Table 4).

##### Type locality.

Abyssal Pacific Ocean, Clarion-Clipperton Zone, 10.32°N, 117.20°W; 4280 m.

##### Etymology.

The species is named in honour of the first author’s younger daughter, Maisie. Used as a noun in apposition.

##### Description.

Based on holotype, immature, NHMUK 2025.33, 8.0 mm. Whole specimen delicate and fragile.

***Body*** (Figs 2, 3): compressed; pereonite 1 longer than 2, 3, or 4. Posterodorsal margins of pleonites 2 and 3, and urosomites 1 and 2 with 5–7 decumbent teeth.

***Head*** (Figs 2, 3): deeper than long; rostrum absent; eye-lobe small, triangular, acute; lateral lobe broadly rounded, lacking notch; eyes absent. ***Antenna 1*** as long as body, peduncle articles 1 and 2 stout; flagellum multiarticulate, nearly 4× length of peduncle; accessory flagellum very small, one article. ***Antenna 2*** about 1/3 length of antenna 1, peduncle articles 4 and 5 slender, subequal, flagellum three-articulate, shorter than peduncle article 5.

***Mouthparts*** (Figs 2, 3, 4, 5): conical. ***Epistome and upper lip*** (Fig. 3): fused, convex, separated by transverse groove; upper lip long. ***Mandible*** (Fig. 5): weakly elongate, molar well developed, not columnar, non-triturative, armed with a crescent of about 20 short, stout spine teeth, long plumose setae on left and right mandibles; accessory setal row with 14 setae, proximal setae simple, distal setae pectinate; incisor process six- and seven-dentate, left and right lacinia mobilis two- and four-dentate; palp article 1 twice as long as broad, about half as long as article 2; article 3 as long as articles 1 and 2 combined, 18 slender setae on oblique distal margin. ***Lower lip*** (Fig. 4): inner lobes prominent, separate, outer lobes narrowly rounded, laterally acute. ***Maxilla 1*** (Fig. 5): inner plate truncate, mesial margin convex, setose; outer plate slender, elongate, stout setae on oblique distal margin; palp as long as outer plate, two-articulate, article 2 about 1.3× length of article 1, 11 stout apical setae. ***Maxilla 2*** (Fig. 5): inner plate broad, subtriangular, facial row of setae set on oblique ridge, mesial margin with pectinate and plumose setae; outer plate slender, setose apically, with transverse cuticular constraint. ***Maxilliped*** (Fig. 4): inner plate truncate with plumose setae; outer plate broad, 11 or 12 stout setal teeth apically and two rows of setae subapically; palp slender, four-articulate, article 2 strongly curved, about 1.5× length of article 3, article 4 much reduced, unguis bifid.

***Pereon*** (Figs 6, 7): ***gnathopod 1*** (Fig. 6): simple; coxa subrectangular, rounded distally, longer than wide, basis long, carpus strong, weakly expanded, 0.5× length of basis, propodus tapering, narrower and shorter than carpus, dactylus strong, as long as propodus. ***Gnathopod 2*** (Fig. 6): subchelate, slender, longer than gnathopod 1; coxa subrectangular, rounded distally, longer than wide, carpus slender, weakly expanded distally, propodus 0.75× length of carpus, palm acute, anterior and posterior margins parallel, posterior margin strongly setose, dactylus bifid distally, closing around single palmar spine. ***Pereopod 3*** (Fig. 7): coxa slightly shorter than coxa 2, posterior margin weakly concave, distal articles slender, merus curved, length about 0.7× length of basis. ***Pereopod 4*** (Fig. 7): coxa sub-square, as long as coxa 3 but wider, anterior margin weakly convex, posterior margin weakly concave, merus curved, length about equal to length of basis and propodus. ***Pereopod 5*** (Fig. 7): coxa bilobate. ***Pereopod 6*** (Fig. 7): coxa posterior lobate, distal margin rounded, basis narrow, margins parallel, length 3.7× width, merus and propodus both longer than basis, dactylus strong, unguiform. ***Pereopod 7*** (Fig. 7): coxa shorter than coxa 6, subrectangular, postero-distal corner rounded, basis narrow, length about 5× width, dactylus unguiform.

***Pleon*** (Figs 2, 3): segment 1 postero-dorsal margin unarmed, segment 2 with five weak teeth, segment 3 with seven acute teeth. ***Epimeron 1*** rounded with weak, obtuse postero-ventral tooth. ***Epimeron 2*** subquadrate, posterior margin convex, small tooth at postero-ventral angle. ***Epimeron 3*** weakly produced, small tooth at postero-ventral angle.

***Urosome*** (Figs 2, 3, 8): segments 1 and 2 with six and five dorso-posterior teeth respectively; segment 3 short. ***Uropod 1*** (Fig. 8): slender, peduncle length 5× width, basofacial setae absent, inner ramus just longer than outer, 0.70× length of peduncle, with dorsal and apical setae. ***Uropod 2*** (Fig. 8): shorter than uropod 1, inner ramus longer than outer. ***Uropod 3*** (Fig. 8): extending beyond uropods 1 and 2, peduncle rectangular, length 2.4× width, short spines on inner margin, rami subequal, slightly longer than peduncle, narrowly lanceolate, long plumose setae on both margins of inner ramus and inner margin of outer ramus, outer ramus with minute second article. ***Telson*** (Fig. 8): small, fragile, 0.40× length of uropod 3 peduncle, cleft 78%, tapering distally, lobes apically rounded with one stout and one slender seta apically and two slender setae dorsolaterally.

##### Sexual dimorphism.

Not apparent.

##### Remarks.

*Mirabestia maisie* sp. nov. is unique among senticaudate amphipods in the possession of conical mouthparts; an elongate and falcate mandible palp article 3; a very slender antenna 2 with a short tri-articulate flagellum; an unusual simple gnathopod 1 in combination with an unusually slender shaped propodus of the subchelate gnathopod 2; coxa 6 strongly posterolobate; coxa 7 deeper than coxa 5; and a disproportionately small and fragile telson.

While *Mirabestia maisie* sp. nov. presents a highly distinct, unusual morphology, molecular barcoding indicated that more than one species was present in the material (Fig. 1). The specimen *Mirabestia* sp. [NHM_6660_AMP1] is clearly separated from *Mirabestia maisie* sp. nov. Owing to the fragility of these specimens, the material of this additional species is not suitable for morphological description at this stage and will be described when sufficient material is available. Although we can see some minor morphological differences between the specimens in the gnathopod 2 propodus (more expanded distally, than in *M. maisie* sp. nov.) we await further material for a full morphological description and provide the barcode and specimen information here to facilitate distinguishing the species.

##### Distribution.

Abyssal Pacific Ocean, Clarion-Clipperton Zone, 4130–4309 m.

##### Molecular data.

Sequence data for the holotype of *Mirabestia maisie* sp. nov. is deposited in GenBank under accession numbers PV077119 (COI) and PV078014 (H3). Sequences of the paratype and additional individuals of the species are deposited in GenBank with the accession numbers as indicated in Table 4. The species has also received a Barcode Index Number from Barcode of Life Data Systems: BOLD: AEB6828 (https://doi.org/10.5883/BOLD:AEB6828).

## Supplementary Material

XML Treatment for Mirabestioidea

XML Treatment for Mirabestiidae

XML Treatment for Mirabestia

XML Treatment for Mirabestia maisie

## Additional information

### Author ORCIDs

Tammy Horton https://orcid.org/0000-0003-4250-1068

Georgina Valls Domedel https://orcid.org/0009-0005-8932-6641

Eva C. D. Stewart https://orcid.org/0000-0001-8383-5705

Michael H. Thurston https://orcid.org/0000-0001-5627-616X

### Data availability

All of the data that support the findings of this study are available in the main text.
